# A biophysically-defined hyaluronic acid-based compound accelerates migration and stimulates the production of keratinocyte-derived neuromodulators

**DOI:** 10.1080/19336918.2018.1494997

**Published:** 2018-08-19

**Authors:** Annalisa La Gatta, Antonella D’Agostino, Chiara Schiraldi, Giuseppe Colella, Nicola Cirillo

**Affiliations:** a Department of Experimental Medicine, Section of Biotechnology, Medical Histology and Molecular Biology, University of Campania “Luigi Vanvitelli”, Naples, Italy; b Multidisciplinary Department of Medical, Surgical and Dental Specialties, University of Campania “Luigi Vanvitelli”, Naples, Italy; c Melbourne Dental School, The University of Melbourne, Melbourne, Australia

**Keywords:** Keratinocytes, directional migration, wound healing, hyaluronic acid, neuropeptides, β-endorfin

## Abstract

Hyaluronic acid (HA) preparations are widely used in clinical practice and recent data suggest that commercially available HA-based compounds promote ulcer re-epithelialization and induce pain relief. However, the pathophysiological basis of these effects remains poorly understood. In the present study, we investigated the biophysical, biomolecular and functional properties of a HA preparation combined with a pool of collagen precursor synthetic aminoacids, namely l-proline, l-leucine, l-lysine and glycine (Aminogam®). Hydrodynamic characterization of Aminogam® by size exclusion chromatography-triple detector array (SEC-TDA) revealed an average molecular weight in the range of 700–1700 kDa. Rheological measurements of the 1700kDa M_w_ lot showed a pseoudoplastic behaviour with a zero-shear viscosity (η_0_) equal to 90 ± 9 Pa∙s at 25°C and 55 ± 6 Pa∙s at 37°C. Automated time-lapse videomicroscopy studies in a fibroblast-free system demonstrated that 1% (v/v) Aminogam® significantly reduced the healing time of wounded keratinocyte monolayers. In AKGOS assays, Aminogam® stimulated cellular locomotion (chemokinesis) and directional migration (chemotaxis) of keratinocytes. Analysis of microarray data suggested that keratinocytes had a functional neuroendocrine machinery, and this was confirmed by testing the secretion of six neuroactive molecules by ELISA, namely α-MSH, β-endorphins, melatonin, substance P, cortisol, and neurotensin. Interestingly, Aminogam® regulated the production of several neuropeptides, including β-endorphins. In conclusion, our data shed light on the epithelial-dependent mechanisms that underlie the efficacy of Aminogam®, particularly in reference to wound healing and nociception.

## Introduction

Hyaluronic acid (HA) is an important constituent of connective tissues and represents a chief component of the extracellular matrix. Commercial preparations based on HA sodium salt (sodium hyaluronate, SH) are widely used in clinical practice, for example in the treatment of osteoarthritis, ocular surgery, and as scaffold for tissue engineering [1]. Recently, we and others have demonstrated that HA-based preparations can act directly on epithelial tissues and are capable of accelerating wound healing in stratified epithelia such as those of the skin and mucous membranes [2–5]. Although it is now well established that HA targets keratinocytes [5] in addition to mesenchymal cells, the mechanisms underlying the putative role of epithelial-derived factors in wound repair have not been fully elucidated.

The successful repair of tissue injury requires a well-coordinated host response that limits the extent of tissue damage [6] while at the same time modulating tissue inflammation and nociception. Interestingly, in addition to promoting epithelial healing, certain HA formulations have been shown to reduce inflammation [7] and pain sensation [8] *in vivo*. In 2001, the US Food and Drug Administration approved a gel based SH as a Class 1 medical device for use in the management of pain relief. Subsequently, results of a pilot study have shown that Aminogam® may be useful for pain management in patients with oral mucositis [2]. This raises the question as to whether HA can stimulate a local neuroendocrine and analgesic activity during the healing process.

An understanding of the biological effects of HA-based compounds cannot be achieved without considering that HA produces different effects depending on its molecular and biophysical characteristics [9]. For example, hyaluronan in its high molecular weight form exhibits anti-inflammatory responses in contrast to low and medium molecular weight forms that stimulate the synthesis of proinflammatory cytokines and chemokines [10,11]. Therefore, it is of paramount importance to determine the biophysical characteristics of HA-based formulations when testing their biological activity.

To shed light on the molecular mechanisms of HA action in keratinocytes, in the present study, we investigated the biophysical (hydrodynamic and rheological), biomolecular (synthesis of neuromodulators), and functional (migration and wound healing) properties of Aminogam®, a commercially available HA preparation combined with a pool of collagen precursor synthetic aminoacids (l-proline, l-leucine, l-lysine and glycine). The results show that Aminogam® accelerates wound healing and promotes the synthesis of several neuroactive molecules, most notably β-endorphins.

## Materials and methods

### Cell culture and reagents

Details of the spontaneously immortalized human skin keratinocyte cell line (HaCaT) have been reported previously [12]. Following transplantation to athymic mice, HaCaT cells are non-tumorigenic and form normal skin [13], therefore are widely used as prototype of normal epidermal keratinocytes. Cells were grown without mesenchymal support in Dulbecco’s modified Eagle’s medium (DMEM) supplemented with 10% foetal bovine serum (FBS) in a humidified atmosphere of 5% CO2/air at 37°C. All reagents for cell culture were from Sigma (Sigma-Aldrich, Gillingham, Dorset, UK). Aminomam® and control HA without amino acids were kindly provided by Professional Dietetics (Milan, Italy).

### Hydrodynamic characterization using a SEC-TDA system (Viscotek)

The SEC-TDA (Size Exclusion Chromatography-Triple Detector Array) equipment by Viscotek (*Viscotek, Malvern, UK*) was used to accomplish a complete hydrodynamic characterization of the sample. A detailed description of the system and its analytical conditions are reported by us elsewhere [14,15]. The sample was diluted about 80fold in water in order to have a column load for each analysis (injection volume × sample concentration × intrinsic viscosity) within the range 0.2–0.4 dl. The molecular weight (M_w_, M_n_, M_w_/M_n_), molecular size (hydrodynamic radius-R_h_), and intrinsic viscosity ([η]) distributions of the sample were derived. The Mark-Houwink-Sakurada (MHS) curves (log [η] vs log M_w_) were also directly obtained [14]. HA concentration in Aminogam*®* was derived by the SEC-TDA analyzes and determined also by means of the carbazole test which is usually employed to quantify HA in marketed formulations [15–17].

### Dynamic viscosity measurements

Rheological measurements were carried out using a Physica MCR301 oscillatory rheometer (Anton Paar, Germany) equipped with a Double Gap cylinder measuring system (measuring bob external and internal diamaters: 26.662 and 24.661mm; measuring cup external and internal diameters 27.583 and 23.824) and a Peltier temperature control.

Flow curves (dynamic viscosity *vs* shear rate) were registered as previously reported [16,18] with slight modifications. Specifically, 50 measuring points were acquired in no time setting mode in the range of shear rate 0.001–1000 s^−1^. Measurements were carried out at 37 and 25°C. From each flow curve, the value of zero-shear viscosity (η_0_, viscosity in the range of Newtonian plateau) was obtained. The η_0_ values reported are the mean value ± SD.

### In vitro keratinocytes scratch assay using time-lapse videomicroscopy (TLVM)

HaCaT cells were used for *in vitro* scratch assay as previously reported [19]. Briefly, 12-wells were seeded with HaCat until complete confluence was reached; scratch wounds were created mechanically with a sterile pipette tip (Ø = 0.1 mm). Uniformly sized scratch was carefully obtained approximately 0.7 ± 0.2 mm in width.

The multi-well was placed in the stage incubator and when the *in vitro* condition was reached, we selected several fields of view for each well and started the time lapse experiment. The ‘wound closure’ phenomenon was monitored for 48h using TLVM station to observe *on line* the phenomena of HaCaT monolayer migrating to repair the wound, in the control and contemporary in presence of Aminogam®. The instrument is equipped with a motorized CO_2_ stage incubator that allows, once selected an image, to return on the same field of view in times defined by the operator [20]. A quantitative analysis of the rate of wound closure was obtained. At each time interval, the percentage of repaired area, the wound closure (%), was calculated as [(Area t_0_ – Area t)/(Area t_0_) × 100] by the software where Area t_0_ represents the area immediately after the wound and Area t represents the area at the time t of the experiment. Each sample was analyzed in triplicate. For each replicate, 6 fields of view were used for deriving the overall averaged curves of wound closure (%) as a function of time thus assuring statistical significance of the experiment. Each field of view (~ 1 × 10^6^ μm^2^) represents 5% of the total scratched area (~ 20 × 10^6^ μm^2^) of each well. Fields of view showing similar scratch width were selected for the analysis.

### RNA extraction and microarray analysis

Evaluation of gene expression in HaCaT cells using microarrays has been reported by us previously [5,21]. Briefly, total RNAs were extracted from cultured cells using TrizolTM solution (Invitrogen), according to the manufacturer’s instructions. The integrity of the RNA was assessed by denaturing agarose gel electrophoresis (virtual presence of sharp 28 and 18 S bands) [22]. Five μg total RNA was used as starting material for the cDNA preparation. The first and second strand cDNA syntheses were performed using the GeneChip One-Cycle cDNA Synthesis Kit (Affymetrix). Labeled cRNA was prepared using the GeneChip IVT Labeling Kit (Affymetrix) according to the manufacturer’s instructions. Following the IVT reaction, the unincorporated nucleotides were removed using RNeasy columns (Qiagen). Fifteen microgram cRNA was used for hybridization onto the Affymetrix Human Genome U133 2.0 probe array cartridge. The washing and staining procedures were performed in the Affymetrix Fluidics Station 450. The probe arrays were scanned at 560nm using a confocal laser-scanning microscope (GeneChip Scanner 3000). The readings from the quantitative scanning were analyzed by the Affymetrix Gene Expression Analysis Software.

### Chemotaxis and chemokinesis (AGKOS) assays

HaCaT keratinocytes were suspended in KGM, counted in a hemocytometer, loaded at a high density (1 × 10^4^ cells per 10 μl) into each 3-mm well in an agarose gel, as detailed elsewhere [23]. In the chemokinesis AGKOS assay, HaCaT were fed with KGM containing various concentrations of test compounds vs no treatment (control) and incubated for 10 days in a humid CO_2_ incubator with daily changes of medium. The migration of keratinocytes was stopped by fixing the cells in 0.25% glutaraldehyde and staining them with Wright’s stain. To measure the effects of the test compound on the random migration distance (RMD) (i.e. the distance outward from the original 3-mm well to the leading edge), the image of each megacolony was projected to the screen and the blueprint obtained. To standardize measurements, three segments were drawn through the center of each megacolony at 60° intervals. The RMD was computed in μm using the following formula:

$$\begin{array}{rl} & RMD=\left(B1B4-A1A4\right)+\left(B2B5-A2A5\right)\\ & \;\;\;\;\;\;\;\;\;\;\;\;\;\;\;\;\;\;\;+\left(B3B6-A3A6\right)/6. \end{array}$$

In the chemotaxis AGKOS assay, HaCaT in KGM were loaded into a 3-mm well in agarose gel, as described above, incubated overnight (to allow cells to settle), after which test HA (50 μl) was inoculated in a 2-mm well cut on one side of the 3-mm well. The incubation was continued for 10 days with daily changes of KGM and HA. After migration was terminated, a blueprint of the outgrowth was obtained and used to compute the directional migration distance (DMD). To standardize measurements, two segments in addition to the median segment B1B4 were drawn through the center of megacolony at the 30° intervals in the direction of the chemoattractant well. The DMD was computed in μm using the following formula:

$$\begin{array}{rl} & DMD=\left(A1B1+A2B2+A6B6\right)-\left(A3B3\right.\\ & \left.\;\;\;\;\;\;\;\;\;\;\;\;\;\;\;\;\;\;\;+A4B4+A5B5\right)/3. \end{array}$$

To control for possible changes of cell cycle progression speed that could affect measurements of migration distances, the effect of Aminogam® on keratinocyte proliferation were assessed separately and no differences (p > 0.05) were found.

### Collection of conditioned media and enzyme-linked immunosorbent assay (ELISA)

Cells were seeded in 6-well plates and grown in complete DMEM until they were 70–90% confluent and then exposed to test compounds as specified in the text. Following experimental treatments, the cells were washed with serum-free media (x3) and PBS (x3) and then incubated in serum-free media for a further 24 hours. The conditioned media (CM) was centrifuged at 2000 RPM for 5 minutes to remove dead cells. The viable attached cells were trypsinized and counted; the CM was normalized for 0.5 x 10^6^ fibroblasts. CM was stored at −20 C°. The production of (α-MSH, β-endorphins, melatonin, substance P, cortisol, and neurotensin) was assessed with Multiplex Human Neuropeptide kit (Millipore, Cat. No. HNP-35K) and quantified with the Luminex® 200™ system (Luminex Corporation, The Netherlands).

### Statistical analysis

Data are given as the average ± SD of independent experiments, as detailed in figure legends. Differences were assessed by *t*-test using pairwise comparisons with corrections for multiple testing and a P value less than 0.05 was considered to be significant.

## Results

### HA content quantification and hydrodynamic characterization using a SEC-TDA system (Viscotek)

**Figure 1.**
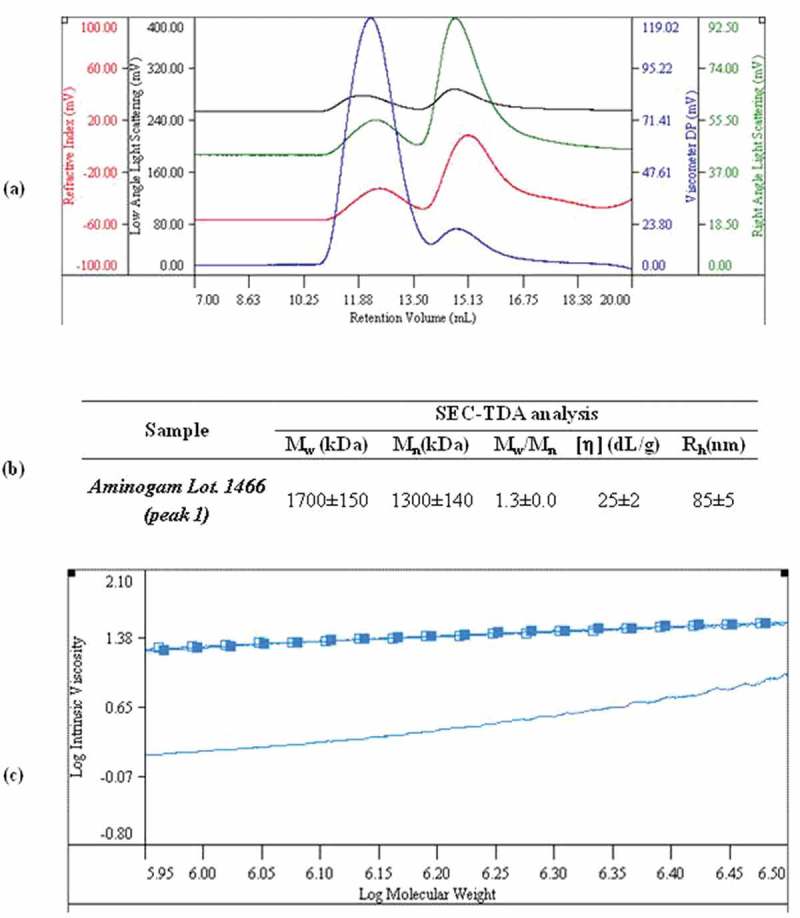
SEC-TDA characterization of *Aminogam®*, lot. N. 1466. (a) Overlap of the chromatographic profiles recorded by the triple detector: the refractive index signal (RI) is in red; laser light scattering signals are in green and black (RALS and LALS, respectively); and the viscosimeter signal (VIS) is in blue. (b) Complete chromatographic report of the first peak: weight average molar mass (M_w_), numeric average molar mass (M_n_), polydispersity index (M_w_/M_n_), intrinsic viscosity ([η]), hydrodynamic radius (R_h_). The analyzes were performed in triplicate (c). Overlap of representative Mark-Houwink-Sakurada curves for a linear HA control (open squares), of the *Aminogam®* sample’ s first distribution (filled squares) and of the *Aminogam®*sample’s second distribution (line).

Chromatographic analyzes of Aminogam® revealed the presence of two distributions that were identified by all the detectors (Figure 1(a)). The distributions were analyzed using the dn/dc value for HA (0.155mL/g) [14]. Results obtained for the first peak are reported in Figure 1(b). A weight average molecular weight equal to 1700 ± 150 kDa and a rather narrow distribution (M_w_/M_n_ = 1.3 ± 0.0) were found. Analysis of other Aminogam® lots revealed that the molecular weight ranged from a minimum of about 700kDa Mw (lot n. 408767) to a maximum value of 1700 kDa (lot n. 1466), with other biophysical parameters varying accordingly (Supplementary Figure 1).

The average intrinsic viscosity and hydrodynamic radius values are consistent with a 1700kDa M_w_ HA sample. The MHS curve (log intrinsic viscosity *vs* log M_w_) related to this distribution was derived and superimposed to the one obtained for a control linear HA sample (M_w_ = 1000 ± 100kDa; M_w_/M_n_ = 1.9 ± 0.2) (Figure 1(c)). The curves perfectly overlap indicating the same intrinsic viscosity for the same molecular weight (same conformation) thus confirming that the macromolecular sample responsible for the chromatographic signal is linear HA [24].

Analysis of the second distribution showed that signals were due to macromolecules other than HA. In fact, as shown in Figure 1(c), the MHS curve for this second distribution does not superimpose to the one of linear HA samples. Specifically, when comparing the same molecular weight values, the intrinsic viscosity of the polymer responsible for this second signal is lower. This indicates a conformation far more compact than the one that HA molecules of the same length are known to exhibit [15,24]. These signals are likely to be due to PVP, which is a component of the formulation as declared by the manufacturer. Based on the SEC-TDA analyzes, the HA content in the 1700kDa M_w_ commercial gel was equal to 11 ± 2 g/L. This concentration value was comparable to the one obtained from carbazole test (p > 0.05, not shown).

### Dynamic viscosity measurements

**Figure 2.**
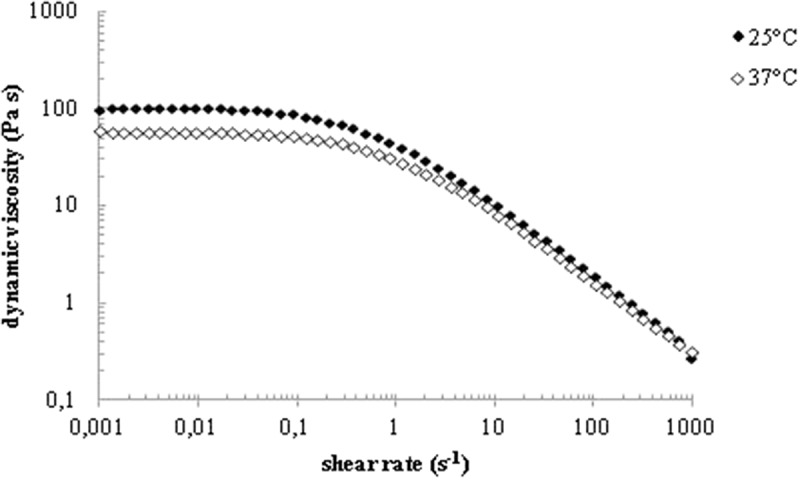
Dynamic viscosity as a function of the shear rate for Aminogam lot.n. 1466. Curves were registered at 25°C and 37°C.

Given that the biological effects and efficacy of a HA-based formulation is partly determined by its rheological properties, we went on to characterize the dynamic viscosity of Aminogam®. Representative flow curves registered at 25 and 37°C for the test compound are reported in Figure 2. An initial Newtonian region in which viscosity remains constant with the shear rate followed by a shear thinning region with viscosity decreasing with shear rate was observed. Such pseudoplastic behaviour is typical of entangled HA solutions [25–27]. The sample’s dynamic viscosity within the Newtonian plateau (η_0_) was equal to 90 ± 9 Pa∙s at 25°C and 55 ± 6 Pa∙s at 37°C. These viscosity values are consistent with the results of the hydrodynamic parameters. Complete characterization was carried out with two additional lots of Aminogam® and the results are reported in Supplementary Figure 1.

### Wound repair in scratched keratinocyte monolayer is accelerated by Aminogam®

**Figure 3.**
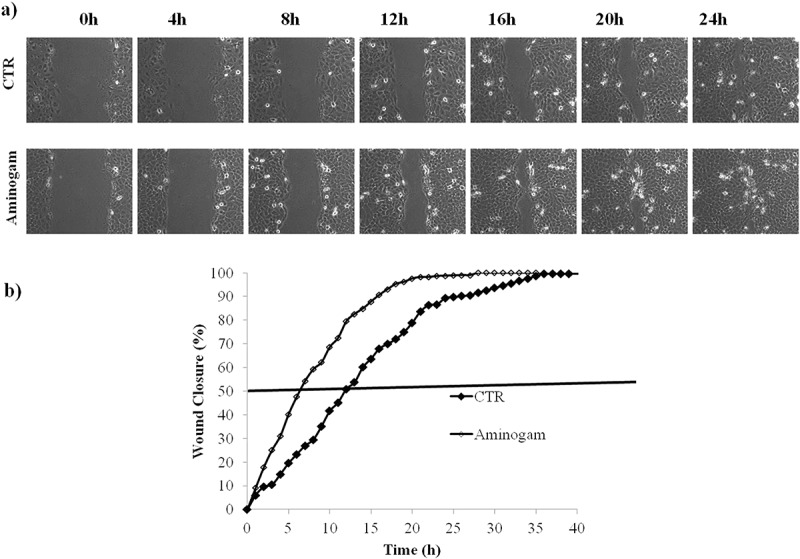
(a) Representative micrographs of HaCaT scratch assays immediately after the scratches, and in time course of the experiments. (b) Repaired area percentage for the control and in presence of Aminogam 1% w/v; the curves are averages of three different experiments with standard deviation within 5% of the value.

Aminogam® treatments were tested in *in vitro* scratch (wound healing) assay using time-lapse videomicroscopy. In preliminary experiments, we determined the optimal concentration of Aminogam® and qualitative assessment of wound healing suggested that keratinocyte migration was accelerated in the presence of 1% (v/v) HA but was impaired at 10% (v/v) (Supplementary Figure 2). As shown in the micrographs (Figure 3(a)), scratched cells treated with 1% (v/v) Aminogam® migrated more quickly compared to untreated control and this effect was seen as early as 4 hours after scratching the monolayer. Quantitative analysis revealed that keratinocytes incubated with the HA-based compound reached 50% closure within 6 hours, whereas untreated cells (control) achieved the same closure after approximately 13 hours (Figure 3(b)). Similar effects were seen with lot 408767 (not shown), which had a lower MW. These data demonstrate that Aminogam® drastically accelerates the migration of keratinocytes *in vitro*.

### Aminogam® promotes random and directional migration of keratinocytes

**Figure 4.**
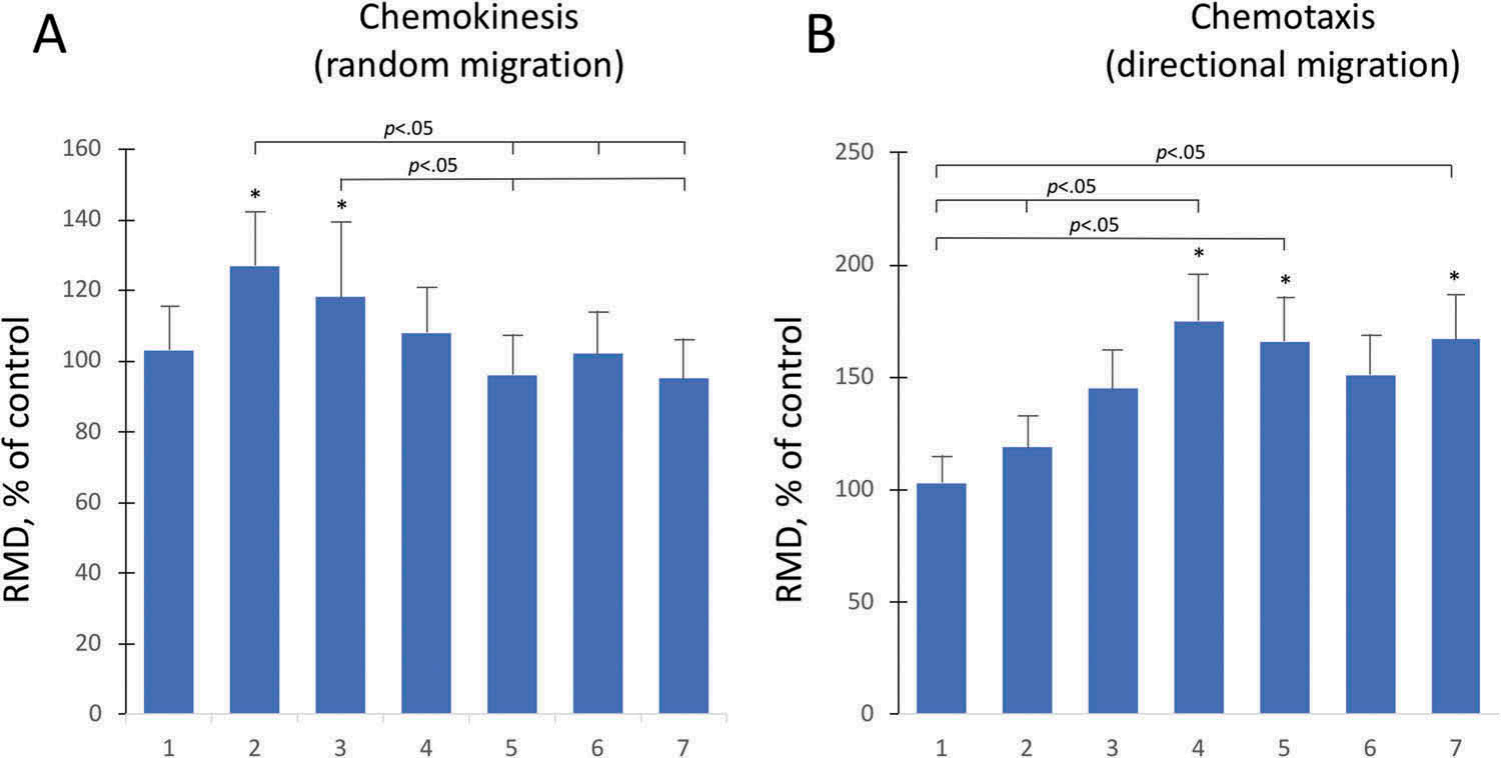
Random and directional migration was assessed with the AGKOS chemokinesis (a) and chemotaxis (b) assays, respectively. The AKGOS plates were monitored for 7 days and the relative migration distance (RMD) was calculated as a % ratio between migration in the presence of serum free medium with (test) or without (control) 1% Aminogam®. Statistical significance refers to test compared to controls at each time point. *, *p* < .05.

The physiological effects of HA on keratinocyte crawling locomotion was investigated in chemokinesis assays. HaCaT were loaded into the chemokinesis AGKOS plates, incubated overnight to allow cells to settle, after which Aminogam® was added at a concentration of 1% (v/v). Random migration appeared to be stimulated at day 2 and day 3, whereas no significant changes were found afterwards (Figure 4(a)). To determine whether aminoacid-enriched HA is chemoattractive for human keratinocytes, we measured directional migration toward Aminogam® using a chemotaxis AGKOS assay. A statistically significant (p < 0.05) increase in the directional migration distance (DMD) was observed starting at day 4 to day 7 (Figure 4(b)). Together, these results show that Aminogam® stimulates cellular motility and acts as a chemoattractant for keratinocytes.

### HA-based compounds modulate the production of keratinocyte-derived neuromodulators

**Figure 5.**
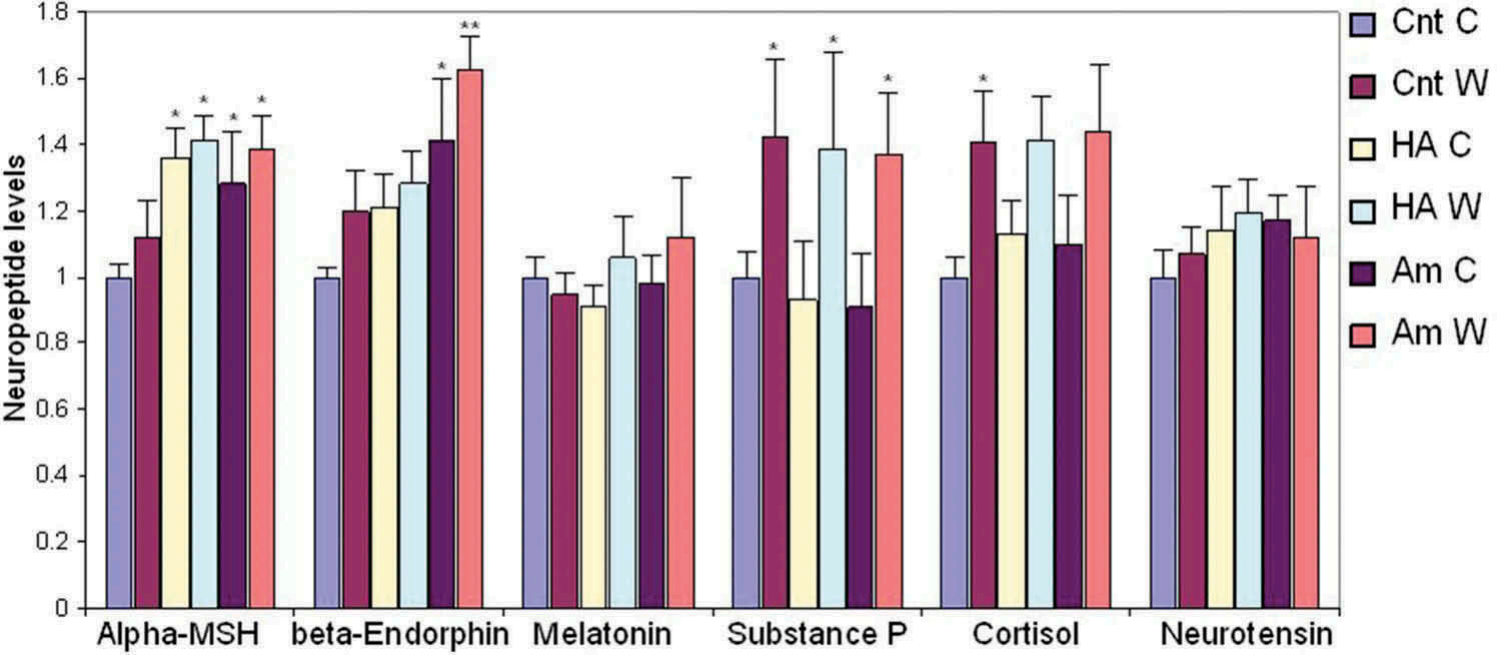
Multiplex ELISA was used to measure the concentration of six molecules in conditioned media from wounded (W) or confluent unwounded (C) keratinocytes cultured in serum-free medium alone (Cnt) or in the presence of either 1% control hyaluronic acid (HA) or a commercial formulation, i.e. Aminogam® (Am). Statistical significance refers to test (HA and Am) compared to controls (Cnt, C or W) at each time point. *, *p* < .05; **, *p* < .01.

It has been reported that Aminogam® reduces inflammation and pain sensation in patients with oral mucositis [2]. To investigate the molecular basis of these observations and to test whether these effects were potentially linked to the neuroendocrine function of the skin, we assessed the production of known neuromodulators (α-MSH, β-endorphins, melatonin, substance P, cortisol, and neurotensin) in keratinocytes. Further, we assessed whether the expression of these molecules was differentially regulated by HA compounds in the supernatant of wounded and unwounded keratinocytes. To investigate whether the main components of the biosynthetic pathway involved in the production of these modulators were expressed by keratinocytes, we analyzed our published microarray data [5]. Gene expression profile revealed that mRNAs encoding for the relevant enzymes and receptors were present in keratinocytes and were modulated to a certain extent by HA preparations (Supplementary Table 1). To confirm that the end-products of these biosynthetic pathways were indeed expressed, we undertook a multiplex ELISA test on culture supernatants. All six neuroactive molecules were secreted by keratinocytes at baseline and expression of α-MSH, β-endorphin, and Substance P was increased either in the presence of HA preparations or during wound healing (Figure 5). Specifically, α-MSH was equally up-regulated by control HA and Aminogam® whereas secretion of β-endorphin was selectively induced by Aminogam®. Production of Substance P increased during wound healing irrespective of HA compounds.

Taken together, these data show that both wounded and unwounded keratinocytes secrete key neuromodulators that are differentially regulated, at least in part, by distinct HA-based preparations.

## Discussion

In the present study, we provide a hydrodynamic and rheological characterization of a commercial HA preparation and demonstrate that this biophysically-defined compound promotes keratinocyte wound healing and secretion of neuroendocrine molecules. In particular, a compound containing a pool of collagen precursor synthetic amino acids (l-proline, l-leucine, l-lysine and glycine) combined with HA (Aminogam®) exhibited selective effects on β-endorphin production compared to control HA (without amino acids).

Chromatographic analyzes revealed that the active biopolymer in the formulation is a high molecular weight HA (700-1700kDa), widely employed in preparations for wound healing purposes [4]. Long HA chains are expected to physically entangle in aqueous medium and this was confirmed here by the viscosity profile of the formulation (Figure 2) showing a pseudoplastic behavior. The dynamic viscosity was high and constant at low shear rates, due to the entangled HA chains occurring under these conditions; then it decreased with the shear rate due to the progressive alignment of the polymeric chains in the flow direction [28]. The physical network formed by the HA chains is expected to act as a porous framework for cell migration, a key feature for a successful healing [29]. It is worth mentioning that such rheological behavior is functional for preparations intended for topical application: the gel flows under the high shear rates occurring during extrusion and, once applied, it remains at the site, due to its high resistance to flow under lower shear rate conditions. Finally, based on the HA molecular weight/concentration values found Aminogam® gel is expected to be highly muco-adhesive.

It is known that HA hastens wound closure of keratinocyte monolayers in *in vitro* scratch wound healing assays [30]. The ability of commercially available HA-based compounds to promote epithelial wound healing has been demonstrated *in vitro* and *in vivo* [5,31]. Here this effect was investigated for the first time using the TLVM technology, the most reliable approach currently available for monitoring 2D-wound healing *in vitro* [32]. In particular, the capability of monitoring cell behaviour within selected areas of the scratch every 1h or less allows to capture the overall migration kinetics within the framework of the experiment. Further, and unlike previous studies, we tested both random and directional migration of keratinocytes.

We have shown recently that high- and low-molecular weight chains of hyaluronan and their hybrid H-HA/L-HA complexes accelerate wound healing of HaCaT cells seeded on collagen-coated plates [19,20]. In this present study, we used uncoated plates to show that Aminogam® promotes keratinocyte migration regardless of integrin-collagen engagement. Because HA interacts with specific proteins in the matrix and with receptors on the cell surface to mediate physiological changes in cells and tissues [33], it is possible that HA in the conditioned medium was sufficient to serve as ECM substrate and to mediate cell-ECM adhesion and locomotion. It would be interesting to assess whether different ECM substrates affect the ability of keratinocytes to migrate in the presence of HA.

An interesting finding of this study is that keratinocytes are capable of producing neuropeptides. Our group has previously reported that keratinocytes synthesize and secrete cortisol [21,34]. Using a non-conventional approach to microarray analysis [21], here we extended these observations to show that HaCaT express an array of enzymes involved in the biosynthesis of neuropeptides such as α-MSH, β-endorphins, melatonin, substance P, cortisol, and neurotensin. Microarrays were performed with RNAs extracted from control keratinocytes and those treated with different types of HA. Synthesis of the end-product (i.e. secreted molecule) was measured in the supernatant by ELISA. To the best of our knowledge, this is the first piece of work reporting the synthesis of substance P and neurotensin by HaCaT keratinocytes. Our data not only reinforce the concept that skin may work as a peripheral neuroendocrine system but, also, provide a mechanistic basis for the clinical effect of HA on inflammation and nociception. A key result of our study was that Aminogam®, but not a control HA compound, increased the production of β-endorfin in wounded and unwounded keratinocytes. This effect was more marked in scratched monolayers, suggesting that the process of wound healing facilitates the synthesis of the opioid neuropeptide. This finding has salient clinical implications, given that β-endorphin is utilized in the body to reduce stress and nociception. This makes Aminogam® a potential treatment option for painful conditions such as skin ulcers and oral mucositis.

It is worth mentioning that the results presented here are related to a specific Aminogam lot, for which a complete biophysical characterization was carried out. However, additional experiments were performed to evaluate potential interlot variations. The results indicated that three diverse preparations present the same HA content while the biopolymer molecular weight varies in a range (700-1700kDa), and a consistent variation in viscosity was registered. Additional time-lapse experiments using the lowest HA molecular weight preparation were performed and a significant increase in the wound repair capacity was still observed. This is in line with previous findings demonstrating that, except for very low molecular weight fragments (< 15kDa), HA fastens the wound healing of human keratinocytes regardless of the molecular weight and that only a slight reduction in the rate of wound closure occurs with the decrease in HA chains length [20]. We conclude that the interlot variation found in Aminogam® influences, to a certain extent, the physical properties related to HA molecular weight (i.e. viscosity), however this does not translate into major variations of the biological effect.

Finally, an important finding of our study is that some of the biological effects induced by Aminogam® are due, at least in part, to the pool of amino acids present in the formulation. Aminogam® and control HA (without amino acids) were found to accelerate wound healing to a similar extent [5]. However, in this present study, we show that there are differences in the biomolecular response of keratinocytes. Specifically, cells incubated with Aminogam®, but not with control HA, secreted increased levels of β-endorphin. The reason for this selective effect is currently unknown. All opioid peptides, including endorphins, are rich in glycine, therefore Aminogam® could provide a substrate for the production of this class of molecules. Alternatively, glycine could act directly or indirectly to activate analgesic mechanisms. In fact, it is known that glycine plays a key role in nociception and that GABA and glycine are often co-localized in the same spinal interneurons and can interact synergistically [35]. Further studies are needed to address these hypotheses.

In conclusion, here we show that keratinocytes exert novel neuroendocrine functions of potential clinical relevance. In particular, we demonstrate that a biophysically-defined HA formulation significantly accelerates keratinocyte migration and selectively enhances the production of the opioid neuropeptide β-endorfin by HaCaT cells. If confirmed by clinical studies, our findings may have a major impact in the treatment of painful conditions such as oral mucositis.

## Supplementary material

Supplementary data for this article for this article can be accesed here.
