# Supplementary figures and images for: A biophysically-defined hyaluronic acid-based compound accelerates migration and stimulates the production of keratinocyte-derived neuromodulators
Source: Cell Adh Migr. 2018 Aug 19;13(1):23–32. doi: 10.1080/19336918.2018.1494997 (PMC6527377; doi:10.1080/19336918.2018.1494997)

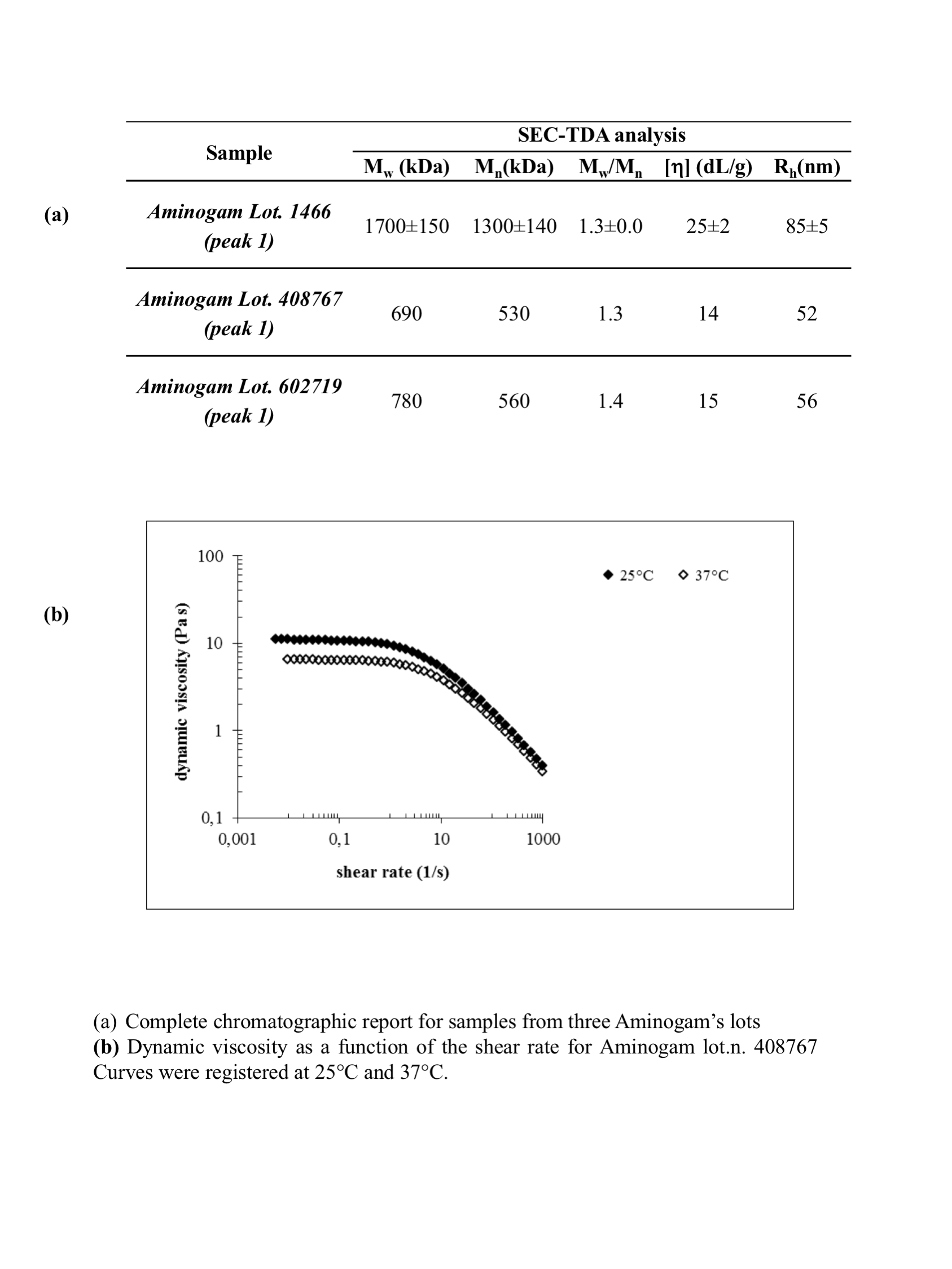

Supplement: Supplemental Material [file kcam-13-01-1494997-s001.zip › Suppl Fig1.tiff]

6

12

24

hrs

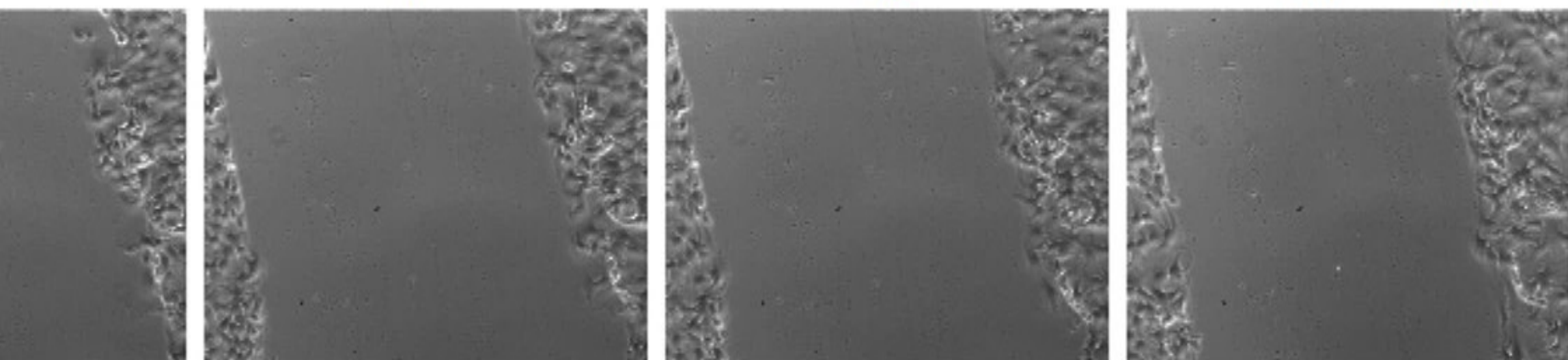

1:10

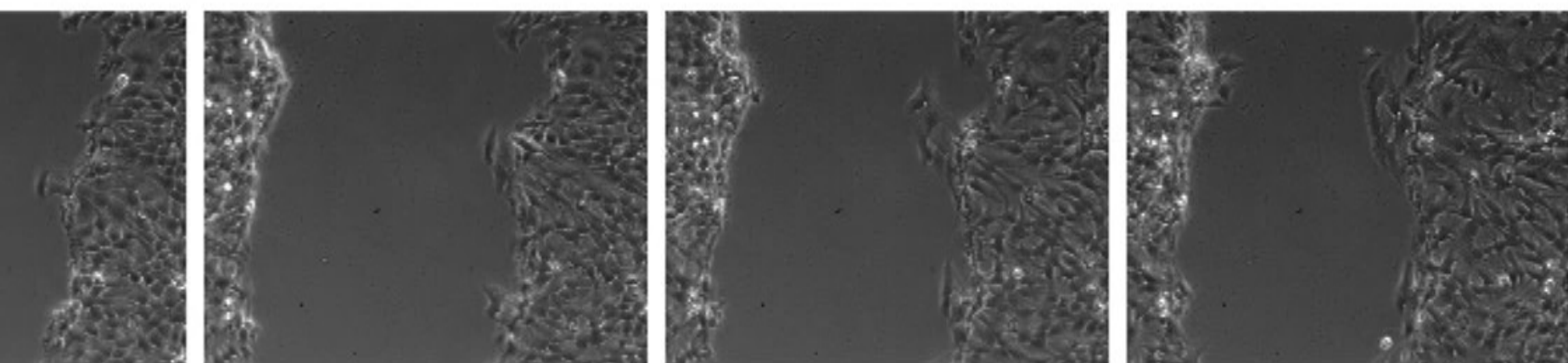

1:100

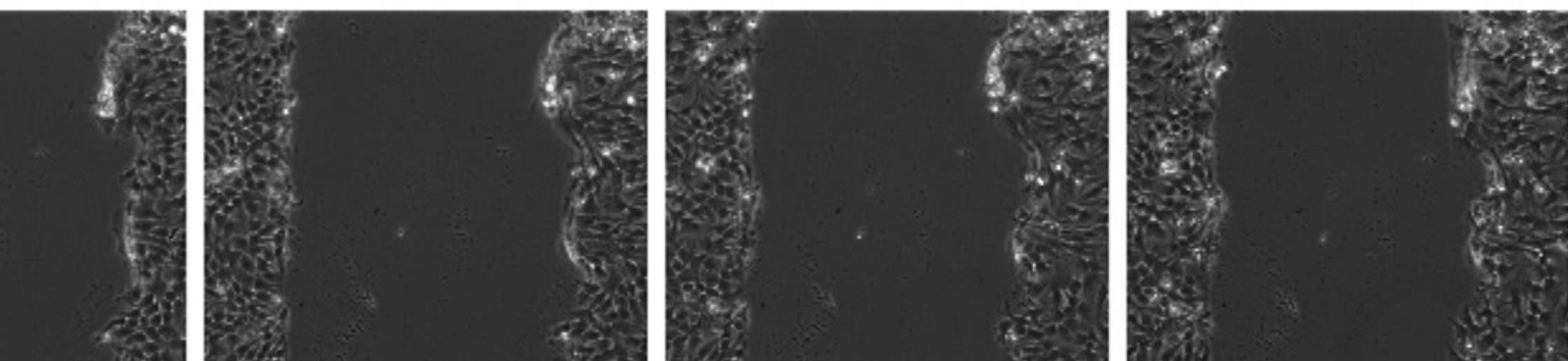

Cnt

Supplement: Supplemental Material [file kcam-13-01-1494997-s001.zip › Suppl Fig2.pdf]
